# Hearing performance of individuals with minimal hearing loss in complex and realistic communication experiences

**DOI:** 10.1590/2317-1782/20232022034en

**Published:** 2023-09-18

**Authors:** Luana Rodrigues do Carmo, Raquel Elpidio Pinheiro da Silva, Cláudia Daniele Pelanda Zampronio, Jerusa Roberta Massola de Oliveira, Maria Fernanda Capoani Garcia Mondelli

**Affiliations:** 1 Hospital de Reabilitação de Anomalias Craniofaciais - HRAC, Universidade de São Paulo - USP - Bauru (SP), Brasil.; 2 Programa de Residência Multiprofissional em Saúde Auditiva, Universidade de São Paulo - USP - Bauru (SP), Brasil.

**Keywords:** Hearing Loss, Hearing Aids, Questionnaires, Performance, Hearing

## Abstract

**Purpose:**

To analyze hearing performance and expectations regarding the use of hearing aids (HA) by participants with minimal hearing loss.

**Methods:**

This research is a primary, observational, longitudinal and prospective study. Two questionnaires, the Speech Spatial Qualities Questionnaire (SSQ) and the Expected Consequences of Hearing Aid Ownership (ECHO), were used, respectively, to verify hearing performance in complex listening situations and expectations regarding the use of HA. The convenience sample consisted of adults aged 53 to 72.

**Results:**

SSQ showed that, for hearing performance, greater difficulties were observed in unfavorable situations such as speech and speech-in-noise, followed by greater ease in locating the sound source and in the quality and naturalness of the sound. ECHO showed that, for the expectations regarding the use of the HA, the variables with significant correlation values were age x general expectation with HA and age x HA's positive aspects. No statistically significant association existed between performance scores in complex listening situations and the analyzed variables.

**Conclusion:**

Minimal hearing loss can negatively influence everyday communicative situations, and the expectation of individuals with minimal hearing loss regarding the use of HA was shown to be high. In addition, the hearing performance of individuals in this study did not show correlations with the age, gender and education level of the sample.

## INTRODUCTION

Hearing loss can be a significant factor in individuals' lives and cause harm to their social interactions and psychological balance and compromise their quality of life^(1)^.

Commonly, hearing loss is classified in degrees related to the sound intensity needed to trigger sound perception in the individual's auditory system^(2)^. However, researchers have suggested a new classification of hearing loss called “minimal hearing loss”^(3)^. This classification is defined in three categories: unilateral sensorineural hearing loss, bilateral mild sensorineural hearing loss and sensorineural loss in high frequencies^(4)^. Minimal hearing loss can become an obstacle to communication in specific situations, such as unfavorable listening situations in environments with noise and reverberation or when there is a great distance between the speaker and the interlocutor^(5)^.

Thus, to improve the hearing skills of individuals with hearing loss, hearing aids (HA) are recognized as one of the therapeutic alternatives and should always be indicated, even in mild hearing loss, to reduce hearing effort^(1)^.

Although the objective data about the HA verification during the rehabilitation process are essential to prove the electroacoustic characteristics, several researchers defend that only the user can determine how well the device solved his need^(6-8)^. Therefore, to verify the HA, the literature has questionnaires that subjectively quantify and qualify, from the user's perspective, the aspects of hearing performance in realistic and complex communication situations and the expectation and satisfaction with HA. In this regard, researchers developed the Speech Spatial Qualities Questionnaire (SSQ), which explores aspects of hearing by measuring the individual's ability to hear speech in different complex listening contexts^(9)^. This instrument demonstrates the benefits of bilateral hearing capacity in different day-to-day situations and different hearing aptitudes^(10)^.

Concerning the qualification and quantification of pre-fitting expectations of individuals who are candidates for HA, researchers have developed the scale known as Expected Consequences of Hearing Aid Ownership (ECHO)^(6)^, which aims to measure the expectations of future HA users, addressing issues such as costs, personal image and expected negative aspects.

Despite technological advancements, HA fitting continues to be a challenge for audiologists, and the high rate of abandonment of the use of HA is an obstacle for health services^(11)^.

Therefore, the present study aimed to analyze the hearing performance and expectations regarding HA use by participants with minimal hearing loss.

## METHODS

This research was approved by the institution's Research Ethics Committee (4,315,301) following the ethical principles of Resolution n° 466/12. This research is a primary, observational, longitudinal, and prospective study. Participants were invited to participate in the research and signed the informed consent form.

Sociodemographic data (age, gender, occupation, education level) and characteristics of hearing loss were obtained through previous analysis of medical records of individuals scheduled to be seen in the sector.

Subjects who met the following eligibility criteria participated in the research:

- Diagnosis of mild bilateral sensorineural hearing loss (average thresholds of 500 Hz, 1000 Hz, 2000 Hz and 4000 Hz greater than 20 dB HL (decibel hearing level) and less than or equal to 40 dB HL, unilateral sensorineural hearing loss (with an average of thresholds greater than 20 dB HL) and hearing loss at high frequencies (thresholds greater than 20 dB HL in frequencies from 2000 to 8000 Hz), as recommended by the National Workshop on Mild and Unilateral Hearing Loss (2005). The degree was established according to the criteria from WHO (1997), as used by the institution;
- Age ranged from 18 to 80 years old;
- No experience with HA.

Subjects who met the following exclusion criteria were removed from the research:

- individuals with cognitive and/or intellectual alterations, identified by the multidisciplinary team's evaluation, that would compromise the data collection.

Two questionnaires were used: the Speech Spatial Qualities Questionnaire (SSQ), which functionally assess hearing difficulties and possible day-to-day difficulties that the participants could present, and the Expected Consequences of Hearing Aid Ownership (ECHO), which qualifies and quantifies the expectations of individuals who are candidates for the use of hearing aids before fitting. Participants answered both SSQ and ECHO on the same day in an interview format.

The collected data were compiled and analyzed using descriptive statistical analysis, with the following parameters: mean, median, confidence interval, standard deviation, minimum, maximum and range. Subsequently, inferential analyses were performed, applying hypothesis tests consistent with the objectives: Student's t-test and Spearman's correlation coefficient. Such hypothesis tests adopted a significance level of less than 0.05 (p = 5%).

As a classification of the degree of correlation, that is, the strength between the variables, the following parameters were used: weak when (0) < r < (0.4), moderate when (0.4) < r < (0.7) and strong when (0.7) < r < (1.0). Correlations had statistical significance when the p-value was less than 0.05, and there was a moderate or strong degree of correlation (12).

## RESULTS

The characterization of the sample had 15 participants with an average age of 62.7 years. The standard deviation for age was 5.95, with a median of 64 years and a range equal to 19. As for gender, 8 females (53%) and 7 males (47%) participated.

**Figure 1:**
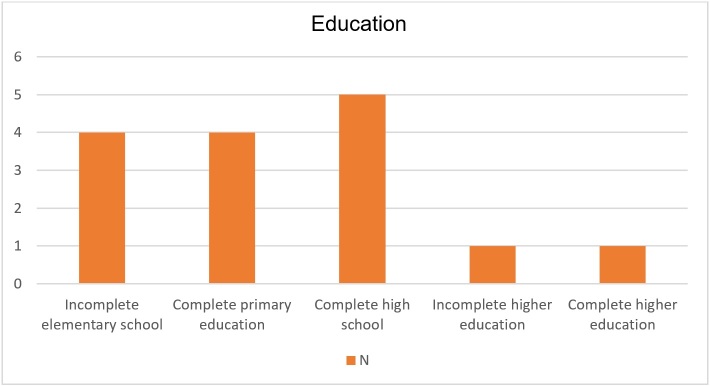
Education variables in the collected sample

As for the schooling variable, completed high school was the most recurrent (n = 5, 33%). Education categories with a long duration of years were the lowest recurrence, with only 1 participant with higher education. Four participants (27%) had incomplete or complete elementary school (Figure 1).

Concerning classifications regarding the type of minimal hearing loss, mild bilateral sensorineural hearing loss was prevalent (n=11, 73%), while only 3 individuals had sensorineural hearing loss at high frequencies (20%), and 1 had mild sensorineural hearing loss in the left ear and high-frequency sensorineural loss in the right.

The SSQ had a lower overall score, with greater difficulty in answering questions 3, 4 and 12, which respectively address the following complex hearing situations: speech-in-speech, speech-in-noise and listening effort. The best answers were obtained in questions 6 (location) and 11 (quality and naturalness). As for the amplitude of the collected data, there was a dispersion of answers with variation between minimum and maximum in four questions (1, 5, 9 and 12), with borderline answers and greater standard deviation for question 9 (segregation). A total of 4.5 per question was obtained in the analysis of means, with a standard deviation of 1.01.

The results obtained are shown in Table 1, and the overall score ranged from 0 to 10 points. Again, a disparity of responses was observed, in the evaluated domains, with discrepant minimum and maximum values.

**Table 1:** Descriptive analysis of the results with the SSQ12 obtained by domains

**Punctuation SSQ12**	**Average**	**Standard deviation**	**Median**	**Amplitude**	**Confidence Interval**	**Minimum-maximum**
**Hearing for speech**	4.2	7.98	4	10	1.80	0-10
**Spatial hearing**	5.4	2.63	7	9	0.76	0-9
**Hearing qualities**	4.18	3.20	5	10	0.80	0-10
**Global**	3.78	2.78	4	10	0.36	0-10

Spearman's correlation coefficient was used to analyze a possible correlation between age/schooling and performance in complex listening situations, and no statistically significant relationships were found in the samples studied for such variables. Student's t-test was used to analyze a possible correlation between gender and hearing performance in complex listening situations, and, again, no statistically significant results were found in the studied sample.

For the ECHO instrument, the following results were collected according to the answers and calculations of the central tendency measures, shown in Table 2. On the one hand, statements 4 and 7, regarding personal image and negative aspects, had the lowest scores, with an average score of around 3. On the other hand, statements 6 and 13, regarding the positive effects of HA and future satisfaction with their model, had the highest average score.

**Table 2:** Descriptive analysis of the results obtained by ECHO

**Affirmative ECHO**	**Average**	**Standard deviation**	**Median**	**Amplitude**	**Confidence Interval**	**Minimum-maximum**
**A1**	5.9	1.19	6	4	0.060	3-7
**A2**	4.2	1.98	5	6	1.00	1-7
**A3**	5.9	1.62	6	6	0.82	1-7
**A4**	3.6	2.03	3	6	1.03	1-7
**A5**	5.6	1.23	5	4	0.62	3-7
**A6**	6.4	0.63	6	2	0.32	5-7
**A7**	3.1	2.07	3	6	1.05	1-7
**A8**	6.3	0.72	6	2	0.36	5-7
**A9**	6.2	0.86	6	2	0.44	5-7
**A10**	4.9	1.92	5	6	0.97	1-7
**A11**	5.9	1.22	6	4	0.62	3-7
**A12**	6.3	0.82	7	2	0.41	5-7
**A13**	6.5	1.06	7	4	0.54	3-7
**A14**	6.2	1.30	5	4	0.66	3-7
**A15**	5.5	1.60	6	5	0.81	2-7

**Caption:** A = Affirmative

As for the overall score, the results are shown in Table 3. The global scale varied with an average of 4.60 to 6.50 points. For the evaluated subscales, the positive effects had the highest average score (5.73), followed by the personal image.

**Table 3:** Descriptive analysis of the results obtained with the global score and between subscales of the ECHO instrument

**Punctuation ECHO**	**Average**	**Standard deviation**	**Median**	**Amplitude**	**Confidence Interval**	**Minimum-maximum**
**Positive Effects**	5.73	0.99	6.00	3.80	0.55	3.20-7.00
**Costs and Services**	5.39	0.82	5.60	2.30	0.45	4.00-6.30
**Negative aspects**	4.47	1.47	4.00	6.00	0.81	3.00-7.00
**Personal image**	5.58	0.19	5.30	2.40	0.44	4.60-7.00
**Global**	5.40	0.45	5.50	1.90	0.25	4.60-6.50

In the analysis of a possible correlation between age and schooling with the expectation regarding the use of HA, statistically significant correlations were found (with a p-value below 0.05 and a correlation coefficient of moderate degree) for the variables age and positive effects, and age and the global ECHO score scale (Table 4).

**Table 4:** Spearman correlation for the expectation regarding the use of hearing aids and the variables age and education

**ECHO**		**R**	***p*-value**
**Age**	**Positive effects**	-0.56	0.029*
	**Costs and services**	0.06	0.828
	**Negative aspects**	-0.24	0.383
	**Personal image**	-0.39	0.155
	**Global**	-0.60	0.018*
**Education**	**Positive effects**	0.17	0.532
	**Costs and services**	0.24	0.374
	**Negative aspects**	0.03	0.923
	**Personal image**	0.46	0.083
	**Global**	0.47	0.073

* Significance level: p <0.05

**Caption:** R = Correlation coefficient

Student's t-test was used to assess the expectation regarding the use of HA obtained with ECHO considering gender, and no statistically significant results were found in the studied sample (Table 5).

**Table 5:** Result of the Student's t-test of the expectation regarding the use of hearing aids considering gender as a variable

		**T-test (p-value)**
**Gender**	**Positive effects**	0.574
	**Costs and services**	0.880
	**Negative aspects**	0.530
	**Personal image**	0.785
	**Global**	0.913

**Caption:** Significance level: p <0.05

## DISCUSSION

After a bibliographic survey in the main databases using keywords in Portuguese and English, little scientific production was found that focused on minimal hearing loss in adults. Consequently, relating the findings of this research with previous studies was difficult. Still, minimal hearing loss can cause adversities in the daily life of affected individuals. Also, the harm caused by mild hearing loss may not be completely measurable only with objective tests, such as pure tone audiometry, but in these cases, using questionnaires and instruments that assess the quality of life of these individuals is the best option to understand their current clinical and functional status^(8)^.

In general, the literature points out listening situations in complex environments, with reverberation, competitive noise and greater distance from sound sources, as major obstacles. Of these situations, those in which speech is present as competitive noise become the most challenging^(9,12,13)^. These data align with the findings in this study since a lower general score was verified in hearing performance, indicating greater difficulty in speech-in-speech, speech-in-noise and listening effort.

Consistent with the unfavorable everyday listening situations most affected by mild sensorineural hearing loss, some studies found that HA users' greatest dissatisfaction is the hearing aid's management regarding background noise, regardless if the noise is caused by speech^(6,14)^. Thus, the capture of sounds that prevented the volunteers from hearing what they wanted to hear had a negative impact even during the hours of daily use of HA.

In the original validation of the SSQ, an overall result of 49 points was obtained in a population of 153 adults and elderly people with bilateral moderate sensorineural hearing loss^(9)^. Similarly, this study found an overall average score of 54 points on the SSQ in a population with minimal hearing loss. Therefore, it is clear that even minimal hearing loss can interfere with hearing performance in complex listening situations since our data are similar to those of the original study. Also, this score corresponds to less than half of the maximum score that can be obtained with SQQ (120).

When analyzing young people with normal hearing, researchers observed a global average of 8.8 in hearing performance in complex listening circumstances^(15)^. In contrast, using the same instrument, the present study generated a global mean response of 4.5. Such low scores may be related to the higher age of the sample and the hearing thresholds, two variables known to cause greater difficulty in daily hearing performance, either due to sensory restriction or cognitive decline^(16)^. Other authors noticed similar performances when comparing young people and middle-aged adults; yet, when there were masking noises, the older individuals experienced substantially more difficulty when the noise had a message in the speech spectrum^(15)^. However, in another study, when evaluating young adults with normal hearing, authors concluded that even those individuals did not report hearing capacity, with great variability of responses^(17)^.

The data collected in this study demonstrated better performances in the domains of location (e.g., localizing a dog barking) and sound quality and naturalness, with the perception of a clear and not cloudy sound. These results align with what is exposed in other national and international studies with different populations, corroborating the design of a profile of hearing abilities more accessible to individuals with minor hearing loss^(9,14,18)^.

As for the domains presented in the SSQ, this study found that the hearing-to-speak and hearing quality factors had the lowest scores, which may mean greater difficulty in tasks related to such domains. Through these answers, audiologists work with different actions during hearing rehabilitation to address the mentioned difficulties better. When aware of the greatest obstacles in the daily life of individuals with minimal hearing loss and with the objective data of the assessment evaluations, audiologists can develop a rehabilitation process that moves towards the singularity needed for a successful HA fitting.

Concerning the expectation regarding the use of hearing aids by the participants, the sample of this study showed results consistent with the literature in general, as high expectations scores were verified^(17,19)^. In fact, for most of the sample, the scores obtained were close to the maximum score of the questionnaire in all sub-items, especially the HA's design and the statement that the HA would “be worth it.” As for the subscale of negative aspects, lower average values of expectation were observed, mainly concerning frustration when the HA detects noises that are unwanted by the user.

Also, a significant relationship was observed between the age variable and the expectation regarding the use of HA among the participants. The increase in age seems proportional to the expectations that the HA will meet all the needs of future users. Unfortunately, this belief can lead to unrealistic ideas about amplification and become an obstacle to the effective use of HA; therefore, greater care is needed in the orientation stage, especially for older individuals.

However, the conclusion that there is a positive relationship between age and the expectation of HA use cannot be taken as definitive in the audiological rehabilitation process since several factors, whether personal or environmental, corroborate the success or failure of the HA fitting^(16,18)^. Indeed, in a systematic review, the authors noted a series of non-audiological factors that could determine the use of HA among the elderly, such as demographic characteristics, the health professional involved in the fitting and the companion of the hearing aid user^(20)^.

Some scholars point out the fine line regarding future HA users' expectations: individuals who do not expect to benefit from the use of HA will not look for them or make optimal use of them; in contrast, those with high expectations may want to try them, but discontinue HA use when they do not provide the expected level of satisfaction^(21)^.

The present study did not demonstrate significant associations (p > 0.05) between gender and education compared with the expectations demonstrated regarding the use of HA or hearing performance in complex listening situations. However, future national studies with the same theme and a larger and more varied sample of participants may lead to new conclusions on the subject.

In general, SSQ's responses had lower scores than those obtained on the ECHO. Therefore, the studied population showed limitations in daily listening environments, mainly in complex listening situations. Such individuals observe and feel these impasses, mainly in communicative situations. A high level of expectation regarding the use of HA was also observed, mainly regarding the positive aspects, which can cause unrealistic expectations and be an obstacle to fitting the HA.

Thus, a service centered on the individual, focused on their needs and particularities, is crucial even in cases of minimal hearing loss. Therefore, this research hopes to corroborate national studies in the area and promote a better understanding of the hearing clinical characteristics of this population in order to maximize their assistance, hearing rehabilitation and quality of life.

## CONCLUSION

Minimal hearing loss can negatively influence everyday communicative situations, and the expectation of individuals with minimal hearing loss regarding the use of hearing aids was high. In addition, the hearing performance of the individuals in this study did not show correlations with the age, gender and education of the sample.
