# Supplementary material for: Hearing performance of individuals with minimal hearing loss in complex and realistic communication experiences
Source: Codas. 2023 Sep 18;35(4):e20220034. doi: 10.1590/2317-1782/20232022034en (PMC10546918; doi:10.1590/2317-1782/20232022034en)
Supplement: Translation

# Translation: Desempenho auditivo de indivíduos com perda auditiva mínima em experiências complexas e realistas de comunicação

**DOI:** 10.1590/2317-1782/20232022034pt

**Authors:** Luana Rodrigues do Carmo, Raquel Elpidio Pinheiro da Silva, Cláudia Daniele Pelanda Zampronio, Jerusa Roberta Massola de Oliveira, Maria Fernanda Capoani Garcia Mondelli

**Affiliations:** 1 Hospital de Reabilitação de Anomalias Craniofaciais - HRAC, Universidade de São Paulo - USP - Bauru (SP), Brasil.; 2 Programa de Residência Multiprofissional em Saúde Auditiva, Universidade de São Paulo - USP - Bauru (SP), Brasil.

**Keywords:** Perda Auditiva, Auxiliares de Audição, Questionários, Desempenho, Audição

## Abstract

**Objetivo:**

Analisar o desempenho auditivo e expectativas quanto ao uso dos aparelhos de amplificação sonora individual (AASI) dos participantes com perda auditiva mínima.

**Método:**

Trata-se de uma pesquisa primária, observacional, longitudinal e prospectiva. Para isso, foram aplicados os questionários *Speech Spatial Qualities Questionnaire* e o *Expected Consequences of Hearing Aid Ownership* que buscam verificar o desempenho auditivo em situações complexas de escuta e a expectativa quanto ao uso de AASI, respectivamente. A amostra se deu por conveniência com indivíduos adultos de 53 a 72 anos.

**Resultados:**

No desempenho auditivo, observaram-se maiores dificuldades em situações desfavoráveis como na fala e fala no ruído, seguidas por maior facilidade em localizar a fonte sonora, qualidade e naturalidade do som. Para as expectativas quanto ao uso do dispositivo de amplificação, as variáveis com valor de correlação significativa foram idade expectativa geral com o uso do AASI, e idade em relação aos seus aspectos positivos. Não houve associação estatisticamente significativa entre os scores de desempenho em situações complexas de escuta e as variáveis analisadas.

**Conclusão:**

A perda auditiva mínima pode influenciar negativamente em situações comunicativas cotidianas, como também, a expectativa dos indivíduos com perda auditiva mínima quanto ao uso do AASI mostrou-se elevada. Além disso, o desempenho auditivo nos indivíduos desse estudo não apresentou correlações com idade, gênero e escolaridade da amostra.

## INTRODUÇÃO

A perda de audição pode ser significativa e ocasionar prejuízo nas interações sociais e no equilíbrio psicológico, bem como comprometer a qualidade de vida do indivíduo acometido^(1)^.

Comumente, a perda auditiva é classificada em graus de comprometimento, relacionados à intensidade de som necessária para desencadear a percepção sonora no sistema auditivo do indivíduo^(2)^. Estudiosos sugeriram uma nova classificação de perda auditiva chamada de ‘’perda auditiva mínima”^(3)^, essa classificação é definida em três categorias, são elas: perdas auditivas sensorioneurais unilaterais, perdas auditivas sensorioneurais leves bilaterais e perdas sensorioneurais em frequências altas^(4)^. Principalmente em ocasiões específicas, a perda auditiva mínima pode se tornar um obstáculo para a comunicação, como em situações desfavoráveis de escuta em ambientes com ruído e reverberação, distância maior entre locutor e interlocutor, entre outros aspectos^(5)^.

Assim, na tentativa de proporcionar a melhora das habilidades auditivas dos indivíduos com perda de audição, o aparelho de amplificação sonora individual (AASI) é reconhecido como uma das alternativas terapêuticas. Esse dispositivo deve ser sempre indicado, mesmo na presença de perdas auditivas de grau leve, visando a redução do esforço auditivo^(1)^.

Diversos pesquisadores defendem que, embora os dados objetivos acerca da verificação do AASI durante o processo de reabilitação sejam essenciais para comprovar as características eletroacústicas na verificação, somente o usuário pode determinar quão bem o dispositivo resolveu sua necessidade^(6-8)^. Para verificação do dispositivo de amplificação sonora, a literatura dispõe de questionários que quantificam e qualificam subjetivamente na visão do usuário, os aspectos do desempenho auditivo em situações realísticas e complexas de comunicação, a expectativa e a satisfação frente ao AASI. Nesse aspecto, pesquisadores desenvolveram o *Speech Spatial Qualities Questionnaire* (SSQ), questionário que explora aspectos da audição mensurando a capacidade do indivíduo para ouvir a fala em diferentes contextos complexos de escuta^(9)^. Esse instrumento, demonstra os benefícios da capacidade auditiva bilateral nas diferentes situações do dia a dia e em diversas aptidões auditivas^(10)^.

No que tange à qualificação e à quantificação das expectativas de pré-adaptação dos indivíduos candidatos ao uso de AASI, pesquisadores desenvolveram a escala conhecida como: *Expected Consequences of Hearing Aid Ownership (ECHO)* ^(6)^. O instrumento visa mensurar a expectativa dos futuros usuários de AASI com seu uso, abordando questões que envolvem assuntos como custos, imagem pessoal e aspectos negativos esperados.

Apesar do avanço tecnológico, a adaptação do usuário de AASI continua sendo um desafio para os audiologistas, e a alta taxa de abandono do uso do dispositivo de amplificação é um entrave para os serviços de saúde^(11)^.

Sendo assim, o presente estudo teve como objetivo analisar o desempenho auditivo e expectativas quanto ao uso dos aparelhos de amplificação sonora individual (AASI) dos participantes com perda auditiva mínima.

## MÉTODO

Trata-se de pesquisa aprovada pelo Comitê de Ética em Pesquisa da instituição (4.315.301), de acordo com os princípios éticos da Resolução n° 466/12. Estudo com delineamento de pesquisa primária, observacional do tipo longitudinal e prospectivo. Os participantes foram convidados a participar da pesquisa e todos assinaram o Termo de Compromisso Livre e Esclarecido (TCLE).

Os dados sociodemográficos foram obtidos por meio da análise prévia dos prontuários: idade, gênero, ocupação e nível de escolaridade dos indivíduos que estavam agendados para serem atendidos no setor, bem como as características da perda auditiva.

Participaram da pesquisa sujeitos que apresentaram os seguintes critérios de elegibilidade:

- Diagnóstico de perda auditiva sensorioneural bilateral de grau leve (média dos limiares de 500 Hz, 1000 Hz, 2000 Hz e 4000 Hz superior a 20 dB Nível de Audição (NA) e menor ou igual a 40 dB NA, perda auditiva sensorioneural unilateral (com média de limiares maior que 20 dB NA) e perda auditiva em altas frequências, com limiares superiores a 20 dB NA nas frequências de 2000 a 8000 Hz, conforme preconiza o *National Workshop on Mild and Unilateral Hearing Loss* (2005). O grau foi estabelecido de acordo com os critérios da OMS (1997), conforme utilizado pela instituição;
- Faixa etária de 18 a 80 anos;
- Sem experiência com o AASI.
Exclusão: indivíduos com alterações cognitivas e/ou intelectuais que comprometam a realização da coleta de dados, identificadas por avaliação da equipe multidisciplinar.

Foram aplicados dois questionários: *Speech Spatial Qualities Questionnaire (SSQ)* para avaliar a dificuldade auditiva de forma funcional, obtendo possíveis dificuldades cotidianas que o participante pudesse apresentar e o instrumento *Expected Consequences of Hearing Aid Ownership* (ECHO), com o objetivo qualificar e quantificar as expectativas dos indivíduos candidatos ao uso de AASI antes da adaptação. Ressalta-se que, tanto a escala ECHO como o questionário SSQ foram aplicados no mesmo dia no formato de entrevista.

Os dados coletados foram compilados e analisados por meio da análise estatística descritiva, com os seguintes parâmetros descritos: média, mediana, intervalo de confiança, desvio padrão, mínimo, máximo e amplitude. Posteriormente, foram realizadas análises inferenciais, com aplicação dos testes de hipótese coerentes aos objetivos: teste T de *Student* e coeficiente de correlação de *Spearman*. Tais testes de hipótese adotaram nível de significância menor que 0,05 (*p* = 5%).

Como classificação do grau de correlação, ou seja, da força entre as variáveis, lançou-se mão dos seguintes parâmetros: fraca quando (0) < r < (0,4), moderada quando (0,4) < r < (0,7) e forte quando (0,7) < r < (1,0). Foram consideradas correlações com significância estatística apenas as que apresentaram valor de p menor que 0,05 e grau de correlação moderado ou forte^(12)^.

## RESULTADOS

A caracterização da amostra contou com 15 participantes com média de 62,7 anos. O desvio padrão para a idade foi de 5,95, com mediana de 64 anos e amplitude igual a 19. Quanto ao gênero, participaram 8 indivíduos do gênero feminino e 7 do gênero masculino, com percentuais de 53% e 47%, respectivamente.

Quanto à variável escolaridade, observou-se como mais recorrente o Ensino Médio completo, com 33% de ocorrência, n = 5. As categorias de ensino com maior duração de anos, como o ensino superior, foram as de menor número, com apenas 1 participante graduado. Para o ensino fundamental incompleto e fundamental completo obteve-se número igual a 4, com percentual de 27% (Figura 1).

**Figura 1:** Apresentação da variável escolaridade na amostra coletada

No que diz respeito às classificações quanto ao tipo de perda auditiva mínima, foi observada prevalência do tipo sensorioneural de grau leve bilateral, com percentual de 73% em 11 participantes, ao passo que apenas três indivíduos possuíam perda auditiva sensorioneural em altas frequências (20%), e um possuía perda auditiva sensorioneural de grau leve em orelha esquerda e perda sensorioneural em altas frequências à direita.

O questionário SSQ, teve uma menor pontuação geral, com maior dificuldade nas respostas das questões 3, 4 e 12, as quais abordam respectivamente as seguintes situações complexas de audição: fala na fala, fala no ruído e esforço de escuta. Em contrapartida, as melhores respostas foram obtidas nas questões 6 (localização) e 11 (qualidade e naturalidade). Quanto à amplitude dos dados coletados, verificou-se dispersão das respostas com variação entre mínimo e máximo em quatro questões (1, 5, 9 e 12) com respostas limítrofes no questionário apresentado, e maior desvio padrão para questão 9 (segregação). Na análise das médias, obteve-se um total de 4,5 por questão, com desvio padrão de 1,01.

Quanto à pontuação geral, os resultados obtidos estão expressos na Tabela 1 e o score global variou de 0 a 10 pontos. Observou-se disparidade de respostas, nos domínios avaliados, com valores mínimos e máximos discrepantes.

**Tabela 1:** Análise descritiva dos resultados com questionário SSQ12, obtidos por domínios

**Pontuação SSQ12**	**Média**	**Desvio Padrão**	**Mediana**	**Amplitude**	**Intervalo de Confiança**	**Mínimo - Máximo**
**Audição para a fala**	4,2	7,98	4	10	1,80	0-10
**Audição espacial**	5,4	2,63	7	9	0,76	0-9
**Qualidades auditivas**	4,18	3,20	5	10	0,80	0-10
**Global**	3,78	2,78	4	10	0,36	0-10

Na análise de uma possível correlação entre a idade/escolaridade e o desempenho em situações complexas de escuta, utilizou-se o coeficiente de correlação de Spearman. Não foram verificadas relações estatisticamente significantes nas amostras estudadas para tais variáveis. Além disso, para a avaliação do desempenho auditivo em situações complexas de escuta obtidas com o SSQ, considerando o gênero, utilizou-se o teste T de Student. Não foram verificados resultados estatisticamente significantes na amostra estudada.

Para o instrumento ECHO, foram coletados os seguintes resultados conforme as respostas e cálculos das medidas de tendências centrais, que se apresentam na Tabela 2. Notou-se como menores scores as afirmativas de número 4 e 7, com pontuação média em torno de 3, relacionadas à imagem pessoal e aos aspectos negativos. Em contrapartida, a afirmativa de número 6 e 13 foi a de maior pontuação média. Estas correlacionam-se aos efeitos positivos com uso do AASI e futura satisfação quanto ao seu modelo.

**Tabela 2:** Análise descritiva dos resultados obtidos por questão do instrumento ECHO

**Afirmativa ECHO**	**Média**	**Desvio Padrão**	**Mediana**	**Amplitude**	**Intervalo de Confiança**	**Mínimo - Máximo**
**A1**	5,9	1,19	6	4	0,060	3-7
**A2**	4,2	1,98	5	6	1,00	1-7
**A3**	5,9	1,62	6	6	0,82	1-7
**A4**	3,6	2,03	3	6	1,03	1-7
**A5**	5,6	1,23	5	4	0,62	3-7
**A6**	6,4	0,63	6	2	0,32	5-7
**A7**	3,1	2,07	3	6	1,05	1-7
**A8**	6,3	0,72	6	2	0,36	5-7
**A9**	6,2	0,86	6	2	0,44	5-7
**A10**	4,9	1,92	5	6	0,97	1-7
**A11**	5,9	1,22	6	4	0,62	3-7
**A12**	6,3	0,82	7	2	0,41	5-7
**A13**	6,5	1,06	7	4	0,54	3-7
**A14**	6,2	1,30	5	4	0,66	3-7
**A15**	5,5	1,60	6	5	0,81	2-7

**Legenda:** A = Afirmativa

Quanto à pontuação geral, foram obtidos os resultados expressos na Tabela 3. A escala global variou com média de 4,60 a 6,50 pontos. Para as subescalas avaliadas, a de efeitos positivos foi a de maior pontuação média, com 5,73, seguida pela escala de imagem pessoal.

**Tabela 3:** Análise descritiva dos resultados obtidos com escore global e entre subescalas do instrumento ECHO

**Pontuação ECHO**	**Média**	**Desvio Padrão**	**Mediana**	**Amplitude**	**Intervalo de Confiança**	**Mínimo - Máximo**
**Efeitos Positivos**	5,73	0,99	6,00	3,80	0,55	3,20-7,00
**Custos e Serviços**	5,39	0,82	5,60	2,30	0,45	4,00-6,30
**Aspectos Negativos**	4,47	1,47	4,00	6,00	0,81	3,00-7,00
**Imagem Pessoal**	5,58	0,19	5,30	2,40	0,44	4,60-7,00
**Global**	5,40	0,45	5,50	1,90	0,25	4,60-6,50

Na análise de uma possível correlação entre a idade e escolaridade com a expectativa quanto ao uso de AASI, foram constatadas correlações estatisticamente significantes (com valor de *p* abaixo de 0,05 e coeficiente de correlação de grau moderado) para as variáveis idade e efeitos positivos, e para idade e a escala global de pontuação do instrumento ECHO (Tabela 4).

**Tabela 4:** Correlação de Spearman para a expectativa quanto ao uso do AASI e as variáveis idade e escolaridade dos participantes

**ECHO**		**R**	***p* valor**
**Idade**	**Efeitos Positivos**	-0,56	0,029*
	**Custos e Serviços**	0,06	0,828
	**Aspectos Negativos**	-0,24	0,383
	**Imagem Pessoal**	-0,39	0,155
	**Global**	-0,60	0,018*
**Escolaridade**	**Efeitos Positivos**	0,17	0,532
	**Custos e Serviços**	0,24	0,374
	**Aspectos Negativos**	0,03	0,923
	**Imagem Pessoal**	0,46	0,083
	**Global**	0,47	0,073

* Nível de significância: p <0,05

**Legenda:** R = Coeficiente de correlação

Para avaliar a expectativa quanto ao uso do AASI obtido com o instrumento ECHO considerando o gênero, utilizou-se o Teste T de Student. Não foram verificados resultados estatisticamente significantes na amostra estudada (Tabela 5).

**Tabela 5:** Resultado do teste estatístico T de Student da expectativa quanto ao uso do AASI considerando a variável gênero

		**Teste T (*p* valor)**
**Gênero**	**Efeitos Positivos**	0,574
	**Custos e Serviços**	0,880
	**Aspectos Negativos**	0,530
	**Imagem Pessoal**	0,785
	**Global**	0,913

**Legenda:** Considerada significância para p <0,05

## DISCUSSÃO

Diante de um levantamento bibliográfico nas principais bases de dados, incluindo palavras chaves em português e inglês, foi observado pouca produção científica voltada para as perdas auditivas mínimas em adultos. Consequentemente, houve dificuldade em relacionar os achados encontrados na presente pesquisa com estudos prévios. Pode-se verificar que a perda auditiva mínima, pode ocasionar adversidades ao cotidiano dos indivíduos acometidos e adicionalmente, é defendido que os danos causados por perdas de audição leves podem não ser completamente mensuráveis apenas com testes, como a audiometria tonal limiar, mas que nesses casos, lançar mão de questionários e instrumentos que avaliem a qualidade de vida desses indivíduos, seja a melhor opção para se compreender melhor o quadro clínico e funcional vigente^(8)^.

A literatura em geral, aponta como maiores entraves, principalmente, as situações de escuta em ambientes complexos, com reverberação, ruídos competitivos e maiores distâncias das fontes sonoras. Dessas situações, aquelas nas quais a fala é presente como ruído competitivo tornam-se as mais desafiadoras^(9,12,13)^. Por conseguinte, estes dados da literatura estão em consonância com os achados no presente estudo, uma vez que foi verificada menor pontuação geral no desempenho auditivo, logo, maior dificuldade nas situações de fala na fala, fala no ruído e esforço de escuta.

Condizente com as situações desfavoráveis de escuta do cotidiano mais afetadas pela perda auditiva sensorioneural leve, alguns estudos verificaram que a maior insatisfação dos usuários de AASI se referia ao gerenciamento do AASI quanto aos ruídos de fundo, sejam eles de fala ou não^(6,14)^. Dessa forma, a captação de sons que impediam os voluntários de ouvir o que queriam, tornou-se impactante mesmo nas horas de uso diário de AASI.

Na validação original do SSQ, foram obtidos um resultado geral de 49 pontos, em uma população de 153 adultos e idosos que possuíam perda auditiva sensorioneural de grau moderado bilateral^(9)^. Enquanto, em nossos achados, mesmo em uma população com perda auditiva mínima, um score geral médio de 54 pontos no questionário SSQ foi encontrado. Logo, faz-se clara a observação de que, mesmo perdas auditivas mínimas, podem acarretar interferências nos desempenhos auditivos em situações de escuta complexas, uma vez que nossos dados se assemelham ao do estudo original e são inferiores à metade da pontuação máxima que pode ser alcançada com o questionário, sendo esta 120.

Pesquisadores, ao analisarem jovens com audição normal, observaram um desempenho auditivo em circunstâncias de escuta complexas, uma média global de 8,8^(15)^. Em contraste, a presente pesquisa gerou uma média global de respostas de 4,5 utilizando o mesmo instrumento. Tal escore rebaixado, pode ter relação tanto com a idade superior da amostra quanto com os limiares auditivos aqui apresentados, duas variáveis que reconhecidamente provocam maior dificuldade na performance auditiva diária, seja pela restrição sensorial ou por declínio cognitivo^(16)^. Outros autores ao comparar jovens e adultos de meia-idade, perceberam desempenhos semelhantes, porém, na presença de ruídos mascaradores, a população mais velha experimentou substancialmente mais dificuldade quando o ruído possuía mensagem no espectro de fala^(15)^. Todavia, em um outro estudo, ao avaliarem jovens adultos com audição normal, chegaram à conclusão de que inclusive indivíduos sem déficit auditivo também não reportaram capacidade auditiva, tendo grande variabilidade de respostas^(17)^.

Os dados aqui coletados, demonstraram melhores desempenhos nos domínios de localização para um latido de cachorro, por exemplo, e para qualidade e naturalidade do som, com percepção de um som claro e não turvo. Estes resultados vão ao encontro do que é exposto em outras pesquisas nacionais e internacionais com variadas populações, pactuando para desenhar um perfil de habilidades auditivas mais acessíveis aos indivíduos com uma perda auditiva menor^(9,14,18)^.

Quanto aos domínios apresentados no questionário SSQ, constatou-se nesse estudo que, tanto o fator audição para falar quanto o fator qualidade auditiva, foram os de pontuação inferiores, o que pode significar maior dificuldade nas tarefas que estejam relacionadas a tais domínios. A partir das respostas adquiridas no instrumento, podem ser levantadas pelo fonoaudiólogo diferentes ações a serem trabalhadas durante a reabilitação auditiva visando melhor alcance das dificuldades referidas. Somando-se as informações de maior entrave no cotidiano de escuta dos indivíduos com perda auditiva mínima, com os dados objetivos das avaliações de verificação, o processo de reabilitação caminha para a singularidade necessária a uma adaptação de AASI bem-sucedida.

Sobre a expectativa quanto ao uso do AASI pelos participantes, a amostra deste estudo demonstrou resultados condizentes com a literatura de modo geral, na medida em que altos escores de expectativas foram verificados^(17,19)^. De fato, para a maioria da amostra, os scores obtidos foram próximos à pontuação máxima do questionário em todos os seus sub itens, com destaque para a aparência do dispositivo e para a afirmação de que o aparato auditivo iria “valer a pena”. Quanto à subescala de aspectos negativos, pode-se notar menores valores médios de expectativa, principalmente em relação à frustração quando o AASI detecta ruídos não desejáveis pelo usuário.

Somado a isso, foi observado relação significativa entre a variável idade e a expectativa quanto ao uso do AASI entre os participantes. Podendo inferir que o aumento da idade é proporcional às expectativas de que o AASI supra todas as necessidades dos futuros usuários. Por conseguinte, essa crença pode culminar em ideias irreais quanto à amplificação e se tornar um empecilho ao uso efetivo e de qualidade do AASI. Portanto, é necessário um cuidado maior na etapa de orientação, principalmente em indivíduos mais velhos.

Contudo, a conclusão de que há relação positiva entre idade e expectativa de uso do AASI não pode ser tomada como definitiva no processo de reabilitação audiológica, uma vez que diversos fatores, sejam eles pessoais ou ambientais, corroboram para o sucesso ou não da adaptação do dispositivo sonoro^(16,18)^. Com efeito, em uma revisão sistemática, os autores notaram que havia uma série de fatores não audiológicos que podiam determinar o uso do AASI entre idosos, dentre os quais destacaram: as características demográficas, o profissional de saúde envolvido na adaptação e o próprio acompanhante do usuário^(20)^.

Alguns estudiosos salientam a linha tênue que existe quanto à expectativa dos futuros usuários de AASI. Para eles, os indivíduos que não esperam se beneficiar com o uso de AASI não os procurarão ou farão o uso ideal deles, ao passo que aqueles com grandes expectativas podem desejar experimentá-los, mas descontinuar o uso quando não proporcionarem o nível de satisfação esperado^(21)^.

O presente trabalho não demonstrou associações de significância (*p*> 0,05) entre gênero e escolaridade em comparação com as expectativas demonstradas quanto ao aproveitamento do AASI, ou mesmo quanto ao desempenho auditivo em situações de escuta complexa. No entanto, mais estudos nacionais com a mesma temática e com amostra maior e variável de participantes podem suscitar novas conclusões acerca do assunto.

Observou-se de modo geral, respostas com escores inferiores no questionário SSQ, se comparado com as respostas obtidas na escala ECHO. Portanto, a população estudada demonstrou possuir limitações em atividades enfrentadas em ambientes de escuta diária, principalmente em situações complexas de escuta. Estes impasses são observados e sentidos por tais indivíduos principalmente em situações comunicativas. Também foi observado nível elevado de expectativa quanto ao uso do AASI principalmente no que tange aos aspectos positivos, o que pode causar expectativas irreais e ser um obstáculo à adaptação do AASI.

Assim, salienta-se a importância de um atendimento centrado no indivíduo, voltado para suas necessidades e particularidades, mesmo em casos de perdas auditivas mínimas. Portanto, a presente pesquisa esperou corroborar com estudos nacionais na área e promover uma melhor compreensão acerca das características clínicas auditivas de tal população no intuito de maximizar sua assistência, reabilitação auditiva e qualidade de vida.

## CONCLUSÃO

Concluiu-se que a perda auditiva mínima pode influenciar negativamente em situações comunicativas cotidianas, como também, a expectativa dos indivíduos com perda auditiva mínima quanto ao uso do AASI mostrou-se elevada. Além disso, o desempenho auditivo nos indivíduos desse estudo não apresentou correlações com idade, gênero e escolaridade da amostra.
